# The association of disproportionately enlarged subarachnoid space hydrocephalus with cognitive deficit in a general population: the Ohasama study

**DOI:** 10.1038/s41598-021-95961-0

**Published:** 2021-08-23

**Authors:** Tomofumi Nishikawa, Ichiro Akiguchi, Michihiro Satoh, Azusa Hara, Mikio Hirano, Aya Hosokawa, Hirohito Metoki, Kei Asayama, Masahiro Kikuya, Kyoko Nomura, Atsushi Hozawa, Naomi Miyamatsu, Yutaka Imai, Takayoshi Ohkubo

**Affiliations:** 1 Faculty of Health Science, Kyoto Koka Women’s University, 38 Kadonocho, Nishikyogoku, Ukyo-ku, Kyoto, 615-0822 Japan; 2 Center of Neurological and Cerebrovascular Diseases, Koseikai Takeda Hospital, Kyoto, Japan; 3 Division of Public Health, Hygiene and Epidemiology, Faculty of Medicine, Tohoku Medical and Pharmaceutical University, Sendai, Japan; 4 Division of Drug Development and Regulatory Science, Faculty of Pharmacy, Keio University, Tokyo, Japan; 5 Department of Human Science, Faculty of Liberal Arts, Tohoku Gakuin University, Sendai, Japan; 6 Department of Occupational Therapy, Faculty of Health Sciences, Aino University, Osaka, Japan; 7 Department of Preventive Medicine and Epidemiology, Tohoku Medical Megabank Organization, Tohoku University, Sendai, Japan; 8 Tohoku Institute for Management of Blood Pressure, Sendai, Japan; 9 Department of Hygiene and Public Health, Teikyo University School of Medicine, Tokyo, Japan; 10 Department of Public Health, Akita University Graduate School of Medicine and Faculty of Medicine, Akita, Japan; 11 Department of Clinical Nursing, Shiga University of Medical Science, Shiga, Japan

**Keywords:** Neurology, Disease prevention, Public health

## Abstract

Disproportionately enlarged subarachnoid space hydrocephalus (DESH) is the characteristic feature of idiopathic normal pressure hydrocephalus. We aimed to characterize the prevalence, development, and association of DESH to cognitive deficit in a large population. We reviewed the data of 1384 subjects eligible for the present study among 1590 participants who underwent magnetic resonance imaging (MRI) in the Ohasama Study, a population-based study in Ohasama, Japan. The participants with Mini-Mental State Examination (MMSE) score <  = 25 were assumed to have cognitive deficit and DESH was evaluated by reviewing the MRIs. We assessed the association between DESH, Evans index (EI), and cognitive deficit using multivariate logistic regression models adjusted for relevant confounders. Furthermore, we evaluated the new development of DESH and the deterioration of cognitive function in the participants with DESH. There were nine participants with DESH (0.65%), seven of whom showed cognitive deficit. DESH was significantly associated with cognitive deficit in multivariate regression analyses (odds ratio; 8.50 [95% confidence interval: 1.61–44.88]). In the 669 participants who underwent follow-up MRI, we found four participants newly presenting with DESH; the development of DESH was observed before/after the presence of EI > 0.3. We also found two participants with existing DESH showing no remarkable worsening in MMSE and EI. The present study demonstrated a positive association between the presence of DESH and cognitive deficit. DESH can develop independently of EI > 0.3, and ventricular enlargement in combination with DESH may be an important factor in the worsening of cognitive deficit.

## Introduction

Idiopathic normal pressure hydrocephalus (iNPH) is known to be a cause of treatable dementia and gait disturbance in elderly patients. For diagnosis of iNPH, the Evans Index (EI) has been widely used to estimate the size of cerebral ventricles^1^, initially described in 1942 as a linear ratio of the maximum width of the frontal horns of the lateral ventricles and the maximal internal diameter of the skull at the same level. Normal values of the EI fall between 0.20 and 0.25, and values above 0.30 indicate definite ventricular enlargement^2^. Consensus guidelines have been accepted that an EI > 0.3 is one of the neuroimaging requisites for the diagnosis of NPH^3^. In addition to an EI > 0.3, when a characteristic pattern of “disproportionate enlargement of the inferior subarachnoid spaces with tight high-convexity subarachnoid spaces” is observed, it is called disproportionately enlarged subarachnoid space hydrocephalus (DESH)^4,5^. The presence of DESH has been proposed to be a potential iNPH-related feature^4^. So far, a few studies have reported the prevalence of DESH^6–8^, and studies on the association between the presence of DESH and cognitive impairment in a general population are still insufficient^9^. We have previously reported that cognitive function in participants with DESH was significantly lower than that in participants with standard magnetic resonance imaging (MRI) findings in a univariate study by observing 506 participants aged 75 years in a community-based birth cohort investigation of the Vienna Trans-Danube Aging study^8^. The present study, therefore, aimed to reveal the prevalence of DESH and its association with cognitive deficit using multivariate logistic regression models in a general population. Furthermore, we followed up the participants and evaluated the new development of DESH along with the change of cognitive function in the participants with DESH.

## Methods

### Design

The present study is a part of the Ohasama Study, which started in 1987 in Ohasama, Japan; the details of this ongoing cohort have been described previously^10,11^. The institutional review boards of Tohoku University School of Medicine, Kyoto Koka Women’s University, and the Department of Health of the Ohasama Municipal Government approved the study.

### Subjects

**Figure 1:**
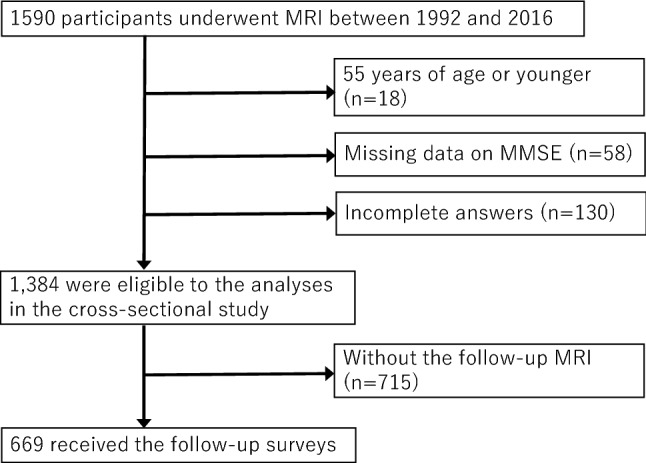
Overview of the study population.

We used data of the participants who provided informed consent and underwent MRI between 1992 and 2016 (n = 1590). Of those, 18 individuals under 55 years of age were excluded because the surveys on MRI and MMSE were mainly performed in the participants over 55 years old in Ohasama study. Moreover, the participants with missing data on the Mini-Mental State Examination (MMSE) (n = 58), and incomplete answers (n = 130). Thus, data of 1,384 participants were eligible for the analyses in the present cross-sectional study (Fig. 1). Among these participants, 669 underwent an MRI and MMSE in the follow-up surveys.

The Japanese version of MMSE was administered to evaluate cognitive function; the details have been described previously^12^. The subjects with a score of <  = 25 were assumed to have cognitive deficit in the present study^13^; in addition, those with scores <  = 26 and <  = 24 were also assessed.

### MRI scanning and evaluation

Images were collected using a Toshiba MRT-50A with a 0.5-T superconducting magnet (Toshiba Medical, Tokyo, Japan)^14^. EI was measured as the ratio of the maximal width of the frontal horns to the internal diameter of the cranium^2^. DESH was diagnosed following the general definition: “disproportionate enlargement of the inferior subarachnoid spaces with tight high-convexity subarachnoid spaces” and “EI > 0.3” on the MRI^4,5^. We sometimes observe “disproportionate enlargement of the inferior subarachnoid spaces with tight high-convexity subarachnoid spaces” in case the EI is 0.29 or so at outpatient clinics. We therefore conducted further analyses to determine if the criterion of EI for diagnosing DESH should be expanded to > 0.28 (“DESH-expanded”). A neurologist (I. A.) and a neurosurgeon (T. N.) independently reviewed MRI recordings from all participants of this study blinded to their clinical information. Disagreement in assessment was resolved by consensus after discussion. The inter-observer reproducibility value for DESH and the kappa coefficient were calculated.

### Statistical analysis

We calculated the odds ratios (ORs) of EI (> 0.3/ =  < 0.3) and DESH (presence/absence) for the prevalence of cognitive deficit using multivariate logistic regression analysis, adjusting for age, sex, history of cerebrovascular diseases (CVD), history of hypertension, history of diabetes mellitus, history of hyperlipidemia, smoking history, drinking history, and duration of education. The duration of education was categorized as being either less than or at least 10 years. We conducted the same analysis on the association between DESH-expanded and cognitive deficit. All significance tests were two-tailed, and p < 0.05 was considered significant in all analyses. All statistical analyses were performed with IBM SPSS Statistics for Windows version 25 (IBM Corp., Armonk, NY).

### Ethics approval

This study conforms to the Helsinki declaration. The Institutional Review Boards of Teikyo University, Tohoku Medical and Pharmaceutical University, and Tohoku University approved the study protocol.

### Consent to participate

Informed consent was obtained from all individual participants included in the study.

## Results

The inter-observer reproducibility value for DESH was 0.99 and the kappa coefficient of the inter-observer reliability was 0.68. There were 92 participants with EI > 0.3 (6.6%), which was significantly associated with age, sex, history of CVD, history of hyperlipidemia, smoking history, and drinking history (Table 1). The prevalence of EI > 0.3 increased with age group, and it was higher in men than in women in all age groups (Table 2). Among the 92 participants with EI > 0.3, nine subjects presented with DESH, so the prevalence of DESH was 0.65% (9/1384) in this population. Seven of them were included in the criteria for cognitive deficit. The presence of DESH was not associated with the relevant variables (Table 1); however, the prevalence of DESH and DESH-expanded tended to increase with age (Table 2).

**Table 1:** Characteristics stratified by Evans index and DESH.

	Evans Index			DESH			Total
	> 0.3	< = 0.3	p value	Presence	Absence	p value	
Number (%)	92(6.6%)	1292(93.4%)		9(0.65%)	1375(99.35%)		1384
Age	68.0 ± 6.1	65.2 ± 6.0	< 0.001	67.8 ± 7.5	65.4 ± 6.0	0.233	65.4 ± 6.0
(Median; range)	(67.7; 55.7–82.5)	(64.9; 55.0–86.0)		(69.4; 55.7–79.7)	(65.1; 55.0–86.0)		(65.1; 55.0–86.0)
Sex (women)	33 (35.9%)	852 (65.9%)	< 0.001	3 (33.3%)	882 (64.1%)	0.055	885 (64.0%)
BMI	23.5 ± 2.7	23.8 ± 3.2	0.342	25.1 ± 2.2	23.8 ± 3.1	0.213	23.8 ± 3.1
History of CVD	10 (10.9%)	65 (5.5%)	0.017	0 (0.0%)	75 (5.5%)	0.471	75 (5.4%)
History of hypertension	52 (56.5%)	605 (46.8%)	0.072	5 (55.6%)	652 (47.4%)	0.626	657 (47.5%)
History of diabetes mellitus			0.503			0.476	
Borderline	7 (7.7%)	138 (10.7%)		0 (0.0%)	145 (10.5%)		145 (10.5%)
Diabetes mellitus	15 (16.3%)	171 (13.2%)		2 (22.2%)	183 (13.4%)		186 (13.4%)
History of hyperlipidemia	10 (10.9%)	288 (22.3%)	0.01	0 (0.0%)	298 (21.7%)	0.115	298 (21.5%)
Smoking history			0.001			0.082	
Current smoker	24 (26.1%)	189 (14.6%)		3 (33.3%)	210 (15.3%)		213 (15.4%)
Former smoker	13 (14.1%)	107 (8.3%)		2 (22.2%)	118 (8.6%)		120 (8.7%)
Never smoker	55 (59.8%)	996 (77.1%)		4 (44.4%)	1047 (76.1%)		1051 (75.9%)
Drinking history			< 0.001			0.39	
Current drinker	46 (50.0%)	433 (33.5%)		5 (55.6%)	474 (34.5%)		479 (34.6%)
Former drinker	7 (7.6%)	33 (2.6%)		0 (0.0%)	40 (2.9%)		40 (2.9%)
Never drinker	39 (42.4%)	826 (63.9%)		4 (60.0%)	861 (62.6%)		865 (62.5%)
Duration of education (< 10 years)	68 (73.9%)	974 (75.4%)	0.751	7 (77.8%)	1035 (75.3%)	0.862	1042 (75.3%)

Continuous data was analyzed using student's t test and is shown in the mean ± standard deviation (median; min.-max.). Categorical data was analyzed using the χ2 test and is shown as number (%).

*MMSE* Mini-Mental State Examination, *CVD* cerevrovascular diseases, *DESH* disproportionately enlarged subarachnoid space hydrocephalus.

**Table 2:** The prevalence of EI > 0.3, DESH and DESH-expanded stratified by age class and sex.

	Age class (y.o.)	55–64	65–74	75 and over	Total
Women	N	438	393	54	885
	EI > 0.3	11	16	6	33
		2.5%	4.1%	11.1%	3.7%
	DESH (EI > 0.3)	0	1	2	3
		0.0%	0.3%	3.7%	0.3%
	DESH-expanded (EI > 0.28)	2	6	4	12
		0.5%	1.5%	7.4%	1.4%
Men	N	241	223	35	499
	EI > 0.3	18	33	8	59
		7.5%	14.8%	22.9%	11.8%
	DESH (EI > 0.3)	3	3	0	6
		1.2%	1.3%	0.0%	1.2%
	DESH-expanded (EI > 0.28)	3	8	0	11
		1.2%	3.6%	0.0%	2.2%
Total	N	679	616	89	1384
	EI > 0.3	29	49	14	92
		4.3%	8.0%	15.7%	6.6%
	DESH (EI > 0.3)	3	4	2	9
		0.4%	0.6%	2.2%	0.7%
	DESH-expanded (EI > 0.28)	5	14	4	23
		0.7%	2.3%	4.5%	1.7%

*EI* Evans index, *DESH* disproportionately enlarged subarachnoid space hydrocephalus (EI > 0.3). DESH-expanded; the criterion of EI for diagnosing DESH was expanded to > 0.28.

The median EI and MMSE were 0.261 (interquartile range, 0.245–0.277) and 28 (interquartile range, 25–29), respectively, in this population. Not only EI > 0.3 but also EI > 0.28 was not associated with cognitive deficit in multivariate logistic regression analyses. In contrast, DESH was associated with cognitive deficit not only in univariate but also in multivariate logistic regression analyses (Table 3). These results were also statistically significant even if the criteria of cognitive deficit were changed (MMSE =  < 24 or =  < 26), or if the criterion of EI for diagnosing DESH was expanded to > 0.28.

**Table 3:** Logistic regression analyses of DESH and Evans index for cognitive impairment in the participants who underwent MRI (n = 1384).

MRI imaging findings			Univariate			Multivariate (model 1)			Multivariate (model 2)		
			OR	95% CI	p value	OR	95% CI	p value	OR	95% CI	p value
MMSE		N									
< = 25/ > 25		373/1011									
EI models											
EI	= < 0.3	343/949	ref			ref			ref		
	> 0.3	30/62	1.33	0.85–2.10	0.207	0.95	0.58–1.55	0.851	0.98	0.59–1.59	0.926
EI	= < 0.28	366/1009	ref			ref			ref		
	> 0.28	7/2	1.62	1.24–2.12	< 0.001	1.19	0.89–1.60	0.231	1.21	0.90–1.63	0.191
DESH models											
DESH DESH	Absence	366/1009	ref			ref			ref		
	Presence	7/2	9.64	1.99–46.65	0.005	7.83	1.50–40.82	0.015	8.50	1.61–44.88	0.012
DESH-expanded	Absence	361/1000	ref			ref			ref		
	Presence	12/11	3.02	1.32–6.90	0.009	2.39	1.01–5.68	0.048	2.60	1.08–6.25	0.032
< = 26/ > 26		497/887									
EI model											
EI	= < 0.3	456/836	ref			ref			ref		
	> 0.3	41/51	1.47	0.96–2.25	0.075	1.05	0.66–1.66	0.833	1.08	0.68–1.71	0.742
DESH model											
DESH	Absence	490/885	ref			ref			ref		
	Presence	7/2	6.32	1.30–30.54	0.022	4.90	0.95–25.25	0.057	5.41	1.04–28.01	0.044
< = 24/ > 24		274/1110									
EI model											
EI	= < 0.3	251/1041	ref			ref			ref		
	> 0.3	23/69	1.38	0.84–2.26	0.197	0.98	0.58–1.65	0.942	1.00	0.59–1.71	0.975
DESH model											
DESH	Absence	269/1106	ref			ref			ref		
	Presence	5/4	5.13	1.37–19.26	0.015	4.07	0.99–16.64	0.051	4.41	1.04–18.60	0.043

Multivariate analyses were performed by adjusting for age, sex, BMI and duration of education (< 10 years) in model 1, and adjusting for history of cerebrovascular diseases, history of hypertension, history of diabetes mellitus, history of hyperlipidemia, smoking history and drinking history in addition to model 1 in model 2.

*95% CI* 95% confidence interval, *EI* Evans index, *BMI* body mass index.

We followed up 669 participants (451 women and 218 men; follow-up period [mean ± s.d.] 8.89 ± 4.55 years, min. 2.6 and max. 24.9), and found 4 participants newly presenting with DESH (two men and two women; mean age: 66.3 years; mean EI, 0.305); the development of DESH was observed before/after the presence of EI > 0.3 (Table 4). Moreover, we also evaluated the deterioration of cognitive function in the participants with DESH in the baseline surveys.

**Table 4:** The development of DESH in the participants who underwent the follow-up MRI (n = 669).

Case	Sex	At the base-line survey			At the latest survey			The interval
		Age	EI	MMSE	Age	EI	MMSE	(years)
1	Woman	61	0.282	30	74.1	0.305	30	13.1
2	Woman	67.7	0.324	21	75.7	0.355	25	8
3	Man	65.3	0.296	30	73.9	0.306	27	8.6
4	Man	71.2	0.322	26	75.2	0.328	23	4

*EI* Evans index, *DESH* disproportionately enlarged subarachnoid space hydrocephalus (EI > 0.3), *MMSE* Mini-Mental State Examination.

We could follow up only two of the nine participants with DESH and found that their MMSE did not change a lot and that their MRI findings were not accompanied by remarkable EI changes either (63.9-year-old man: MMSE 21–20 and EI 0.317–0.324 for 4 years; 55.7-year-old man: MMSE 27–27 and EI 0.355–0.335 for 7.2 years).

## Discussion

To our knowledge, the present study is the most extensive one that demonstrates the prevalence of DESH and the development of DESH in a general population. Moreover, we found that DESH was positively associated with cognitive deficit using multivariate logistic regression analyses, adjusting for relevant variables. In the follow-up surveys, we found that DESH developed independently of existing EI > 0.3, and it did not necessarily accompany the decline of cognitive function. Besides, the follow-up surveys demonstrated individuals with existing DESH showing no remarkable worsening in MMSE; in these cases, EI did not increase a lot.

As DESH is diagnosed based on an EI > 0.3, EI is the index essential for diagnosing ventricular enlargement in clinical sites. However, the mean EI is considered variable depending on the studied population, i.e., it is influenced by differences in age, gender, and related variables, while it is still controversial^15^. The proportion of the participants with EI > 0.3 (6.5%) in the present study was similar to that in many studies (2.8%: mean age, 59 years^15^; 16.1%: mean age, 75 years^8^; 17.0%: mean age, 73.6 years^16^; 6.5%: 61 and 70–72 years^6^), and the mean EI was also reasonable since it was compatible with other population-based studies^16–18^, excluding one study (mean EI, 0.248 ± 0.022: mean age, 70.6 years)^1^. Following these results and ours, it is appropriate to consider that EI increases with age and is greater in men than in women^16,19^. The same applies to DESH. The prevalence of DESH, limited to 75 years and over, in the present study (2.2%; 2 in 89 participants) was consistent with that in other studies (1.5–2.0% at 61–79 years)^6–8^. The present study demonstrated an age-dependent increase in the prevalence of the “DESH-expanded” when the criterion of EI for diagnosing DESH was expanded to > 0.28. Besides, our follow-up surveys demonstrated the development of DESH. These findings strongly suggest age-dependent increases in the prevalence of DESH.

In the present study, EI was not found to be associated with cognitive deficit after sex differences and age-dependent changes being taken into consideration. On the other hand, in a recent survey on 314 residents (mean age, 70.6 ± 7.9; mean EI, 0.246 ± 0.022; EI > 0.3 was seen in six subjects, 1.9%), there was a significant inverse relationship between EI and cognitive function in multivariate linear regression analyses^1^. The difference between these two studies is unclear, but the difference in the mean EI mentioned above might have influenced the results. In contrast, while DESH is diagnosed based on an EI > 0.3, the presence of DESH was independently associated with cognitive deficit in the present study. The difference in the association to cognitive deficit between EI and DESH may be explained by the assumption that DESH contains more specific pathological information than EI. In the present study we didn’t evaluate other indexes like z-Evans index, which is defined as the maximum z-axial length of the frontal horns of the lateral ventricles to the maximum cranial z- axial length and is thought to have a high affinity to DESH^21,22^, such indexes may positively associate with cognitive deficit.

As shown in Table 4 and DESH-expanded cases, “disproportionate enlargement of the inferior subarachnoid spaces with tight high-convexity subarachnoid spaces” developed before/after EI exceeded 0.3. DESH is thought to be induced by perivascular space narrowing, particularly at the centrum semiovale^8^, which is considered to be associated with cerebral amyloid angiopathy in cortical and leptomeningeal arteries^20^. These pathological changes should occur independently of an EI > 0.3; therefore, it may be reasonable to consider that the development of “disproportionate enlargement of the inferior subarachnoid spaces with tight high-convexity subarachnoid spaces” was observed before/after EI exceeded 0.3. In other words, whether EI exceeds 0.3 or not may depend on the complex of various cerebrospinal fluid hydration pathway obstructions, including perivascular space narrowing.

It is also unclear whether asymptomatic DESH is a risk for cognitive deficit or not. Iseki et al. have reported that 8 out of 12 individuals with DESH were asymptomatic, and 2 of them developed dementia and/or gait disturbance with worsening of ventriculomegaly during the follow-up period of 4–8 years^6^. In contrast, while we could follow up only two of the nine participants with DESH, neither of them showed a remarkable change in MMSE; at the same time, EI did not increase a lot. These findings may suggest that ventricular enlargement, in combination with the emergence of DESH, plays an essential role in the deterioration of cognitive function.

The present study has some limitations. First, the number of participants with DESH might be too small to conduct multivariate logistic regression analyses after adjustment for some relevant variables. Second, the present study did not assess gait disturbance, which is also one of the most critical symptoms of iNPH. Finally, the DESH was qualitatively identified following its characteristic pattern; therefore, the kappa coefficient of the inter-observer reliability in the present study may be insufficient for the diagnosis of DESH for clear identification, and the development of universal grading systems, like the Fazekas scale^23^, may be needed.

## Conclusion

The present study is the most extensive one on the prevalence of DESH and the development of DESH, involving the observation of 1384 participants in a general population and demonstrating that the presence of DESH was associated with cognitive deficit using multivariate analyses. In contrast, the follow-up study showed that the development of DESH did not always accompany cognitive deficit and that DESH without a remarkable increase in EI did not cause the deterioration of cognitive function. These findings may suggest that DESH develops independently of an EI > 0.3 and the emergence of ventricular enlargement in combination with disproportionately enlarged subarachnoid space plays an essential role in the worsening of cognitive function in DESH.
